# A 15-year bibliometric analysis of body mass index moderation in the management of Parkinson’s disease

**DOI:** 10.3389/fnagi.2025.1614719

**Published:** 2025-09-26

**Authors:** Qiying Liu, Ying Chen, Shuyi Chen, Tao Qiu, Ziyi Guo, Yuqiang Lu

**Affiliations:** ^1^First Clinical Medical College, Zhejiang Chinese Medical University, Hangzhou, Zhejiang, China; ^2^Department of Rehabilitation Medicine, The First Affiliated Hospital of Zhengzhou University, Zhengzhou, China; ^3^National Engineering Laboratory for Internet Medical Systems and Applications, The First Affiliated Hospital of Zhengzhou University, Zhengzhou, China; ^4^Department of Neurology, Tongde Hospital of Zhejiang Province Afflicted to Zhejiang Chinese Medical University (Tongde Hospital of Zhejiang Province), Hangzhou, China

**Keywords:** Parkinson’s diseases, body mass index, bibliometric, CiteSpace, VOSviewer

## Abstract

**Objective:**

Parkinson’s disease (PD) is a chronic progressive neurodegenerative disorder characterized by worsening motor symptoms, that significantly impair patients’ activities of daily living and quality of life. Emerging evidence indicates a correlation between body mass index (BMI) and PD progression and prognosis. However, few systematic bibliometric analysis has been conducted in this research field. This study aims to comprehensively investigate the clinical translation potential and research hotspots of BMI management in PD pathogenesis, treatment, and care through bibliometric approaches.

**Methods:**

Publications related to BMI and PD research from 2010 to 2024 were retrieved from the Web of Science Core Collection. Bibliometric analysis was performed using “VOSviewer” “CiteSpace” and the R package “bibliometrix.”

**Results:**

A total of 588 publications from 267 journals were analyzed. Parkinsonism and Related Disorders ranked as the most productive journal with 33 publications. Analysis of regional research contributions indicates that the United States, China, and Italy demonstrate predominant scientific influence in this discipline. Among 3,707 contributing authors, Barichella M emerged as the most co-cited author, with research spanning PD-BMI correlation, nutritional management, and gut microbiota. Keyword cluster analysis (e.g., “weight gain” “weight loss” “body mass index” “nutritional management”) highlighted metabolic alterations and nutritional interventions as research focal points, underscoring the role of BMI fluctuations in PD progression.

**Conclusion:**

This study presents the first systematic bibliometric summary of clinical translation research on BMI modulation in PD management. The findings enhance understanding of BMI’s critical role in PD research, provide theoretical foundations for BMI-based interventions to delay disease progression and reduce mortality, and identify novel research directions to advance PD treatment toward a multidisciplinary collaborative model.

## 1 Introduction

Parkinson’s disease (PD) is a progressive neurodegenerative disorder characterized by loss of substantia nigra pars compacta dopaminergic neurons (Dong-Chen et al., 2023), and the patient’s ability to take care of himself declines rapidly as the disease progresses. Epidemiological studies indicate that the global prevalence of PD is 200/100,000 people, and it is estimated that the number of PD patients will double by 20,409 (Leite Silva et al., 2023).

PD presents with a broad spectrum of clinical manifestations encompassing both motor and non-motor symptoms. The motor dysfunctions primarily include the clinical manifestations of PD encompass a wide range of motor dysfunctions, including abnormally rigidity, slowed movement speed, resting tremor, and postural and balance disorders, accompanied by a variety of non-motor symptoms. These symptoms involve sleep disorders, emotional anxiety and depression, cognitive impairment, and impaired autonomic regulation. Both motor and non-motor characteristics such as bradykinesia, stiffness, tremor and postural instability can interfere with food intake and dietary composition (Fereshtehnejad et al., 2014). A study from the German Federal Statistical Office statistically found that Body Mass Index (BMI) was reduced in patients with advanced PD (Bachmann et al., 2009). Weight loss is a common symptom of PD and is associated with decreased quality of life (Wills et al., 2017), Kacprzyk et al. (2022) published an article suggesting that 1/3 of PD patients are at risk of malnutrition, which has become one of the risk factors for poorer quality of life and health outcomes in PD patients (Sheard et al., 2014). A peek at the research trends summarizing the impact of BMI modulation in PD patients on their disease progression may be instructive for those who want to conduct research in this area.

Reviews are based on qualitative analysis and rely on authors’ subjective interpretation and synthesis of the literature to summarize the current state of research and provide insights. Bibliometrics processes literature data through statistical and mathematical models to reveal research trends, hotspots, and impacts, allowing one to analyze a large number of publications and their production patterns at both macro- and micro-levels (Kokol et al., 2021). The Web of Science database is one of the most suitable databases for bibliometric analysis due to its inclusion of peer-reviewed high-quality research papers and its good reputation in the academic community (Ma et al., 2021). In this paper, we use bibliometric methods to quantitatively analyze the citations of scientific papers based on the construction of citation maps. In recent years, there has been a gradual increase in the number of bibliometric research papers in the field of medicine, providing a comprehensive overview of the research field and driving researchers to identify trends, hotspots, and gaps in the literature (Mayr and Scharnhorst, 2015). A literature review of studies on the correlation between BMI and PD is available, and no bibliometric analyses have yet been conducted. Upon searching, we found that the research in this area can be traced back to the early 2000s, and in order to comprehensively examine the research trends, we selected 2010–2024 as the period of research.

## 2 Methods

### 2.1 Data source and search strategies

**Figure 1:**
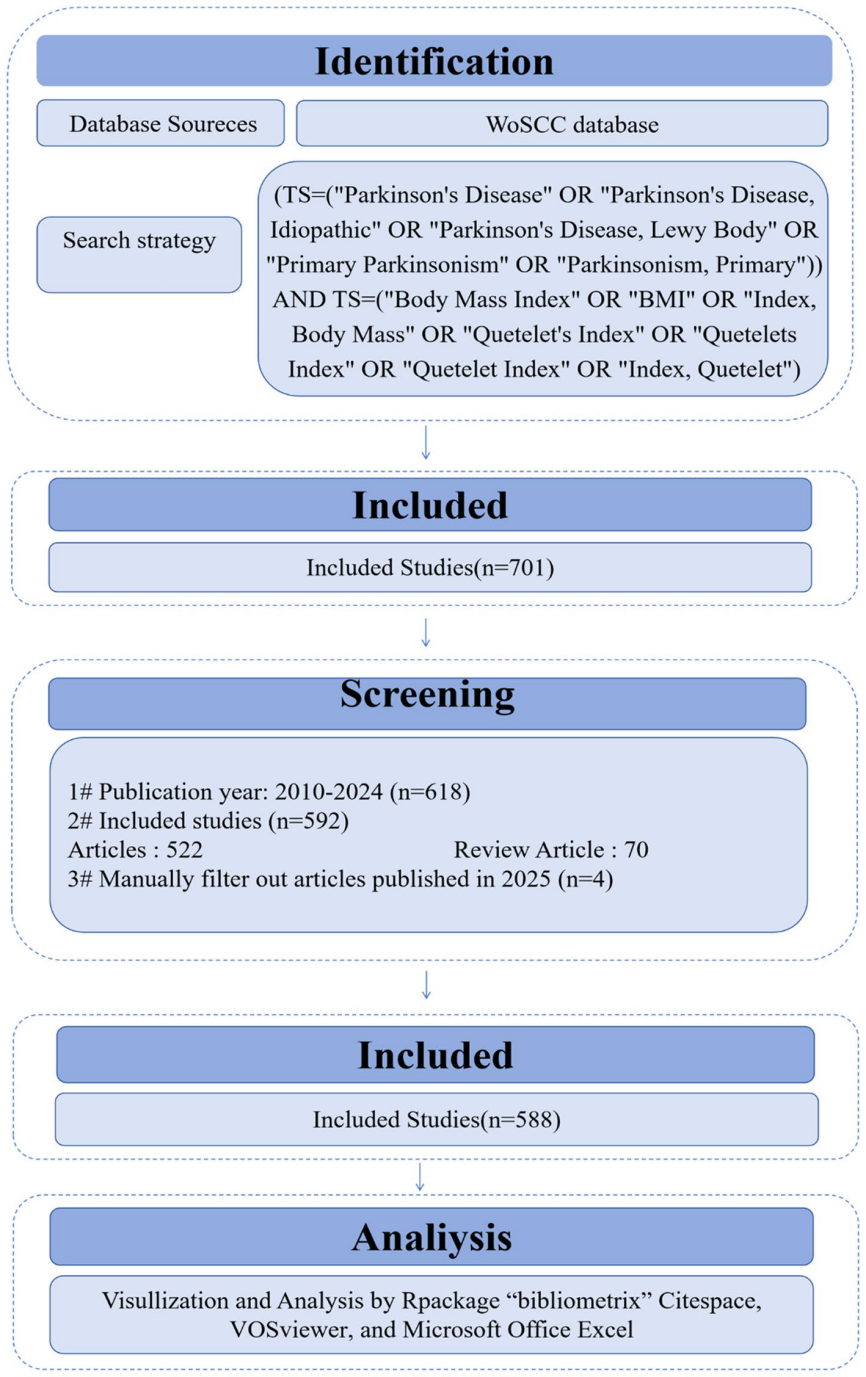
Flowchart of publication search and selection.

This paper provides an econometric analysis for the literature published from 2010 to 2024 to examine the relationship between BMI and PD. Other databases were excluded due to overlapping with the Web of Science. Focusing on a single source ensures efficient data processing while providing a comprehensive view of the relevant literature and avoiding the redundancy that may arise from using multiple similar databases. The established the Web of Science search formula was as follows: (TS = (“Parkinson Disease” OR “Idiopathic Parkinson Disease” OR “Idiopathic Parkinson’s Disease” OR “Lewy Body Parkinson Disease” OR “Lewy Body Parkinson’s Disease” OR “Paralysis Agitans” OR “Parkinson’s Disease, Idiopathic” OR “Parkinson’s Disease” OR “Parkinson’s Disease, Idiopathic” OR “Parkinson’s Disease, Idiopathic” OR “Parkinson’s Disease, Lewy Body” OR “Primary Parkinsonism” OR “Parkinsonism, Primary”)) AND TS = (“Body Mass Index” OR “BMI” OR “Index. Body Mass” OR “Quetelet’s Index” OR “Quetelets Index” OR “Quetelet Index” OR “Index, Quetelet”). Inclusion criteria: (1) The type of literature was a collection of papers or a review; (2) Publication year: 2010–2024; (3) Language: English; (4) Abstracts or full texts were read by three researchers (LQY, CY and CSY) and screened individually (Figure 1). Exclusion criteria: editorial material, retracted publications, book chapters, corrections, conference abstracts, conference papers, early access articles, letters, retractions, and publications expressing concerns.

### 2.2 Data analysis and visualization

This study analyzed the obtained data in the following five aspects: (1) scientific output analysis: annual scientific output volume, scientific output volume of countries and journals, and their trends. (2) Collaboration analysis: including author, country/region and faculty/institution collaborations. (3) Co-occurrence analysis: identifying co-occurring keywords, authors, research institutions and countries. (4) Co-citation analysis: including co-cited authors, co-cited documents and co-cited journals. (5) Emergence analysis: including citation emergence analysis and keyword emergence analysis.

In this paper, we used the software “CiteSpace 6.4 R1 (32-bit) (Drexel University, America)” “Bibliometrix package for R language (University of Naples Federico II, Italy)” “VOSviewer (Centrum voor Wetenschap en Technologische Studies, Netherlands)” “Excel (Microsoft Corporation, America)” “Tableau Desktop 10.5 (Tableau Software, America)” to analyze the indexed literature data to present the above five scientometric analyses and their visualization. Figure 1 illustrates the analytical approach of the study.

## 3 Results

### 3.1 Annual publications and trends

The average annual number of publications from 2010 to 2015 (budding period) is below 30, with a slow growth rate (Compound Annual Growth Rate of about 12%).

**Figure 2:**
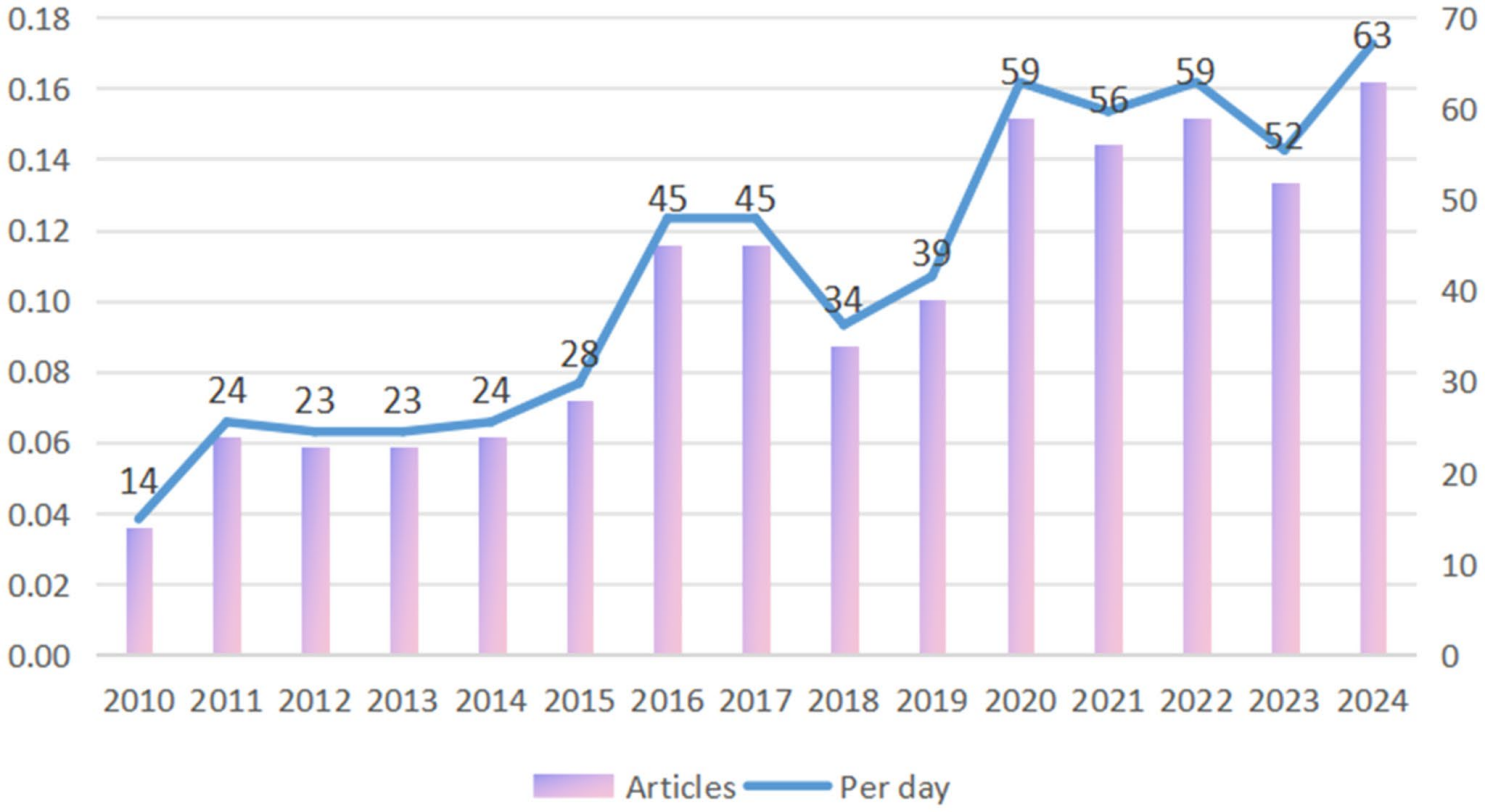
Annual scientific production on research in BMI and PD from 2010 to 2024.

During the growth period of PD and BMI research from 2016 to 2020, the number of papers published will jump to 45–59, with a compound annual growth rate about 20%, a significant increase of the growth rate, and its research is mainly focused on method development and proof of principle. The peak of 59 in 2020 may reflect the outbreak of COVID-19 pandemic-driven virological, vaccinological, epidemiological, and telemedicine research. The neurophilic nature of COVID-19 has the potential to exacerbate chronic neurological diseases, including PD. The potential mechanisms underlying this exacerbation include systemic inflammatory responses and pharmacodynamic changes, such as interactions between norepinephrine and the renin-angiotensin system in the substantia nigra and striatum (Cilia et al., 2020). By 2021 to 2024, the number of publications stabilizes at a high level (52–63), with a slower growth rate than before, but with a sustained number of publications per day higher than 0.14/day (Figure 2).

### 3.2 Analysis of the countries

**Figure 3:**
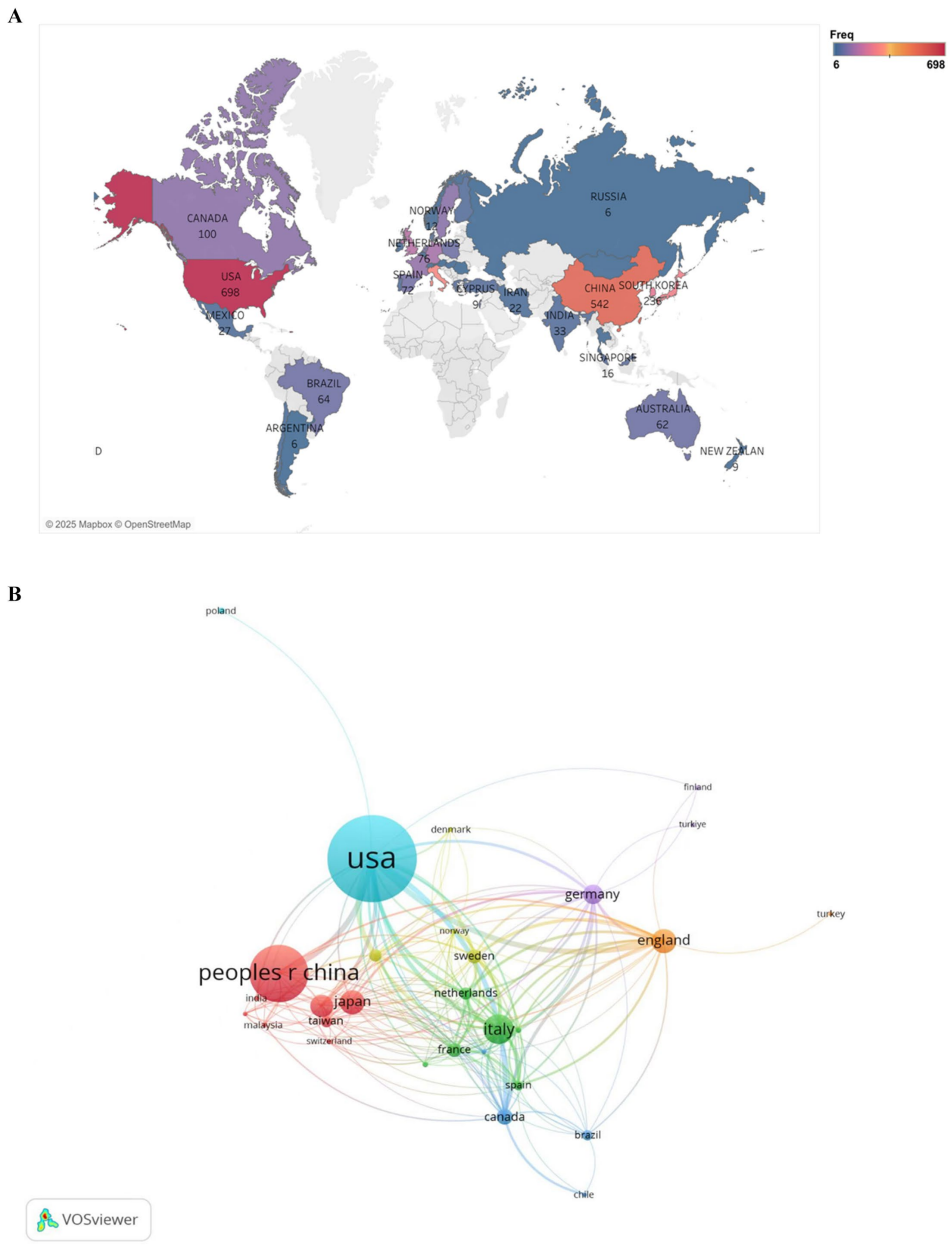
Geographic distribution of national publications frequency **(A)** and visualization of countries **(B)** for research on BMI and PD. **(A)** Created with Tableau Desktop 10.5; **(B)** Created with VOSviewer.

Figure 3A displays the scientific output of countries in the field of PD and BMI research, including only those with a publication frequency greater than five. In the figure, regions shaded closer to red indicate a higher publication frequency, while those closer to blue represent a lower frequency. The United States leads globally in publication volume, followed by China. The high output of the United States is closely tied to its robust research funding infrastructure and extensive clinical resources. Among European nations, the Netherlands, Spain, and the United Kingdom demonstrate notable productivity, whereas Nordic countries such as Norway show relatively limited contributions.

Figure 3B illustrates the collaborative networks among countries using a network diagram. Larger node sizes indicate higher publication volumes, with node size reflecting each country’s centrality in the collaboration network. Node colors distinguish between different collaboration patterns, while the thickness of connecting lines represents the strength of partnerships. As shown, the nodes for the United States and China are the largest and connected by the thickest line, underscoring their central roles in international collaborations and their strong bilateral partnership. Additionally, the United States maintains robust collaborations with countries such as Canada, the United Kingdom, and Australia, reflecting its extensive global research network.

Global research on BMI and PD exhibits a highly uneven distribution, with traditional leaders like the United States maintaining dominance while the Asia-Pacific region experiences rapid growth. Moving forward, fostering balanced development in this field will require enhanced international cooperation and optimized resource allocation.

### 3.3 Analysis of the international cooperation

Table 1 presents national-level data on publication volume, citation frequency, and collaboration patterns in BMI and PD research. The United States and China jointly lead in publication output (111 articles each), yet demonstrate marked disparities in research impact: U. S. publications accumulated significantly higher total citations (5,737) compared to China’s 2,066. Italy, despite a modest publication count (42 articles), exhibited exceptional research quality with 3,834 total citations and a remarkable average of 91.3 citations per article – substantially exceeding the U. S. (51.7) and China (18.6). Japanese research demonstrated unique collaborative characteristics (MCP = 0), with all 38 publications produced domestically without international collaboration, potentially reflecting distinctive national research practices. In contrast, the United Kingdom (MCP = 12) and Sweden (MCP = 6) showed stronger international collaboration tendencies while maintaining outstanding research impact (47.1 and 94.1 average citations per article, respectively).

**Table 1:** Top 10 countries by research output and collaboration patterns in BMI and PD.

Country	Article	SCP	MCP	Total citations
USA	111	96	15	5,737
CHINA	111	89	22	2066
ITALY	42	33	9	3,834
KOREA	39	31	8	754
JAPAN	38	38	0	896
UK	27	15	12	1,272
GERMANY	21	14	7	362
FRANCE	17	12	5	422
SWEDEN	16	10	6	1,505
BRAZIL	15	13	2	420

SCP, Single country publication; the quantity or proportion of SCP reflects the strength of a country’s domestic research collaboration and its independent research capability. MCP, multiple country publication. The quantity or proportion of MCP reflects the extent of a country’s involvement in international research collaboration and its level of globalization.

**Figure 4:**
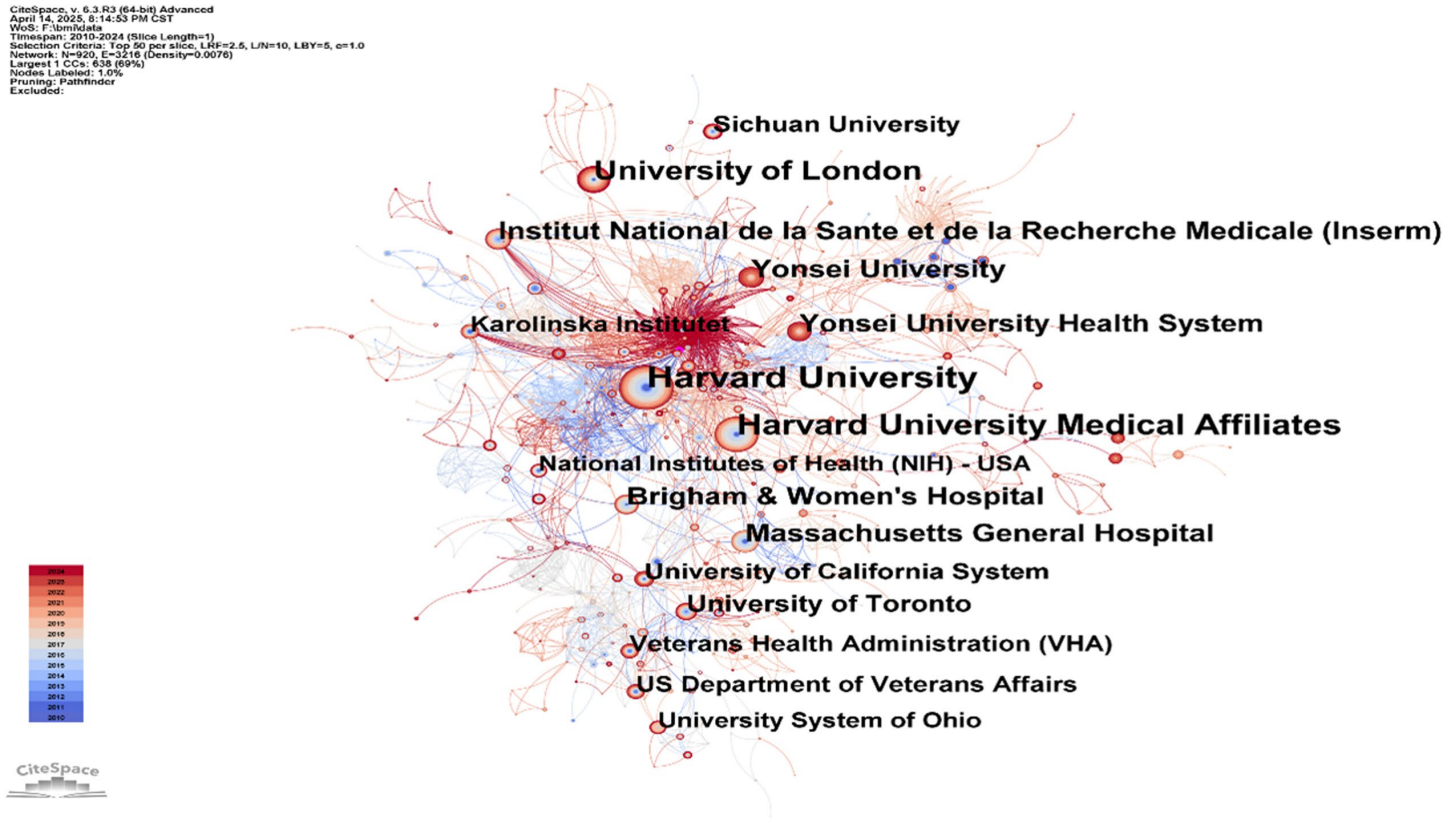
Institutional collaboration map: BMI and PD research. Created with CiteSpace 6.4 R1.

Table 2 show the top-ranked countries studying BMI and PD and their paper publications. Analysis of institutional contributions shows that the top 10 institutions are located in seven different countries, reflecting the prevalence of the impact of BMI on aspects of the condition and treatment of PD. The United States and China lead the field of research (111 papers). Yonsei University (South Korea) is the most prolific institution with 62 publications, followed by University of Malaya (Malaysia) with 31 publications, and Soochow University in this country with 30 publications. Other key institutions include Rush University (USA), Umea University (Sweden), etc. Karolinska Institute (Sweden), McGill University (Canada), Sichuan University (China), and Harvard University (USA) also made significant contributions with 24–28 publications each. Figure 4 illustrates the collaborative network of global research institutions in this field. The different colored clusters in the figure indicate the distribution of various research directions and fields, highlighting the centrality of institutions such as Harvard University, Harvard University Medical Affiliates, Massachusetts Gen Hospital, Yonsei University, University of London. In addition, the figure illustrates the collaborative relationships between these institutions and other research entities. International collaboration has demonstrated significant enhancement of research impact, though notable regional disparities persist in current collaborative patterns. Consequently, future efforts should focus on optimizing collaborative networks, improving research quality, and facilitating knowledge translation to promote balanced development in this field.

**Table 2:** Top 10 institutions on research of BMI and PD.

Institutions	Articles
Yonsei University	62
University of Malaya	31
Soochow University	30
Rush University	29
Umea University	29
ASCHERIO	28
Karolinska Institute	28
McGill University	26
Sichuan University	25
Harvard University	24

### 3.4 Analysis of the journals and co-cited journals

From 2010 to 2024, research on BMI and PD was published across 267 journals. As presented in Table 3, the top 10 most-cited journals were led by Parkinsonism and Related Disorders (33 articles), followed by Frontiers in Neurology (17 articles) and Movement Disorders (17 articles). And other high-impact journals included the Journal of Parkinson’s Disease and PLOS ONE.

**Table 3:** Top 10 journals and co-cited journals for research on BMI and PD.

Sources	Count	Source	co-citations
PARKINSONISM AND RELATED DISORDERS	33	movement disord	2004
FRONTIERS IN NEUROLOGY	17	neurology	1,491
MOVEMENT DISORDERS	17	parkinsonism relat d	959
JOURNAL OF PARKINSONS DISEASE	15	plos one	516
PLOS ONE	14	j neurol neurosur ps	505
NEUROLOGY	13	ann neurol	484
NEUROLOGICAL SCIENCES	12	lancet neurol	364
FRONTIERS IN AGING NEUROSCIENCE	11	arch neurol-chicago	340
JOURNAL OF THE NEUROLOGICAL SCIENCES	10	brain	329
JOURNAL OF NEUROLOGY	9	am j epidemiol	326

**Figure 5:**
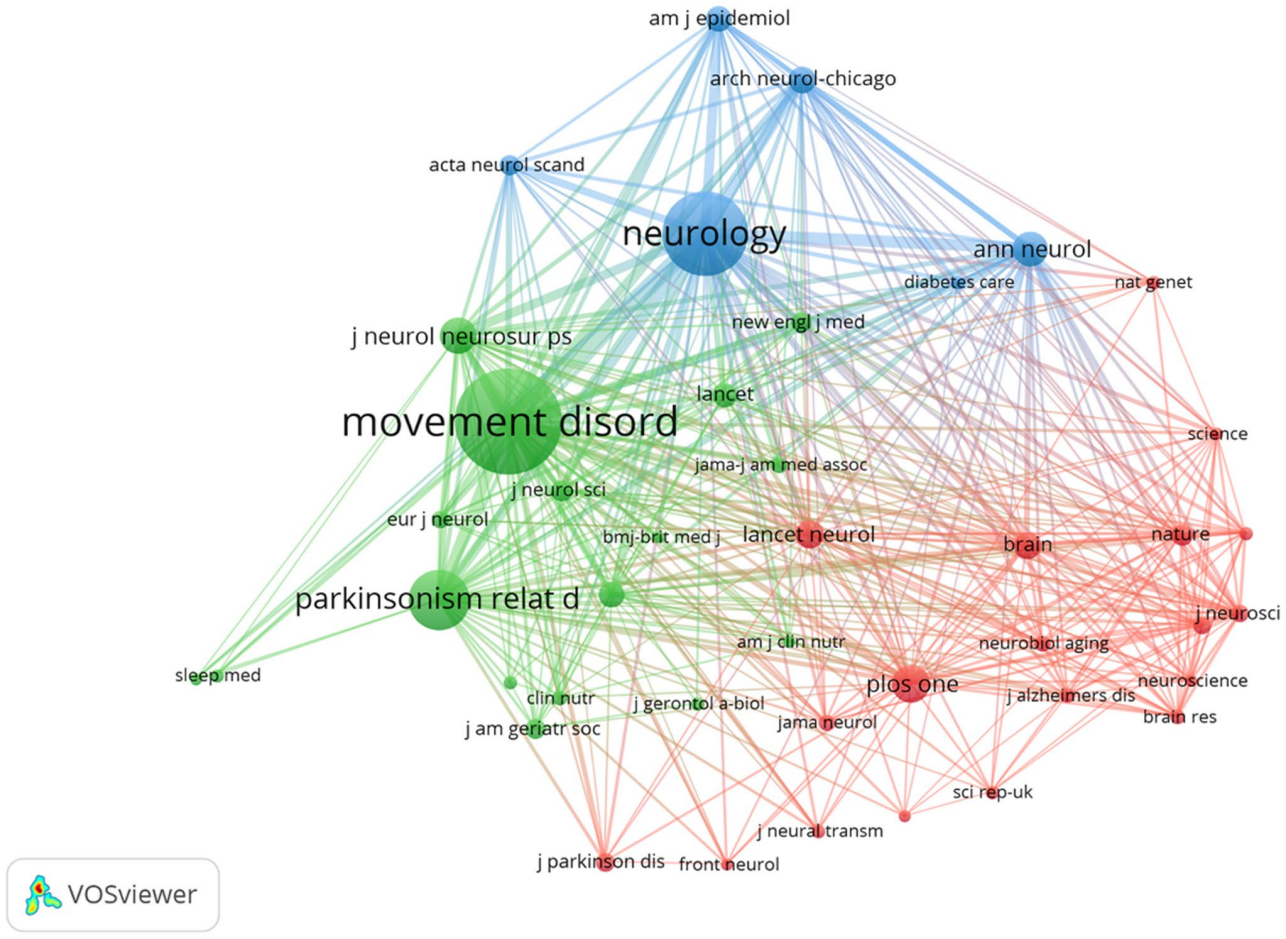
The visualization of journals for research on BMI and PD. Created with VOSviewer.

Figure 5 illustrates a citation network with journals as nodes. The purple cluster represents journals primarily focused on basic research, while the green cluster encompasses those emphasizing biomedical and applied studies. Among the most cited journals, Movement Disorders ranked first with 2,004 citations, followed by Neurology (1,491 citations) and Parkinsonism and Related Disorders (959 citations).

Notably, the frequently co-cited journals exhibited substantially higher impact factors, with The New England Journal of Medicine and The Lancet achieving the highest metrics within this group. These publications are widely recognized as peer-reviewed, long-established, and high-impact journals in the field.

### 3.5 Analysis of the authors and co-cited authors

**Figure 6:**
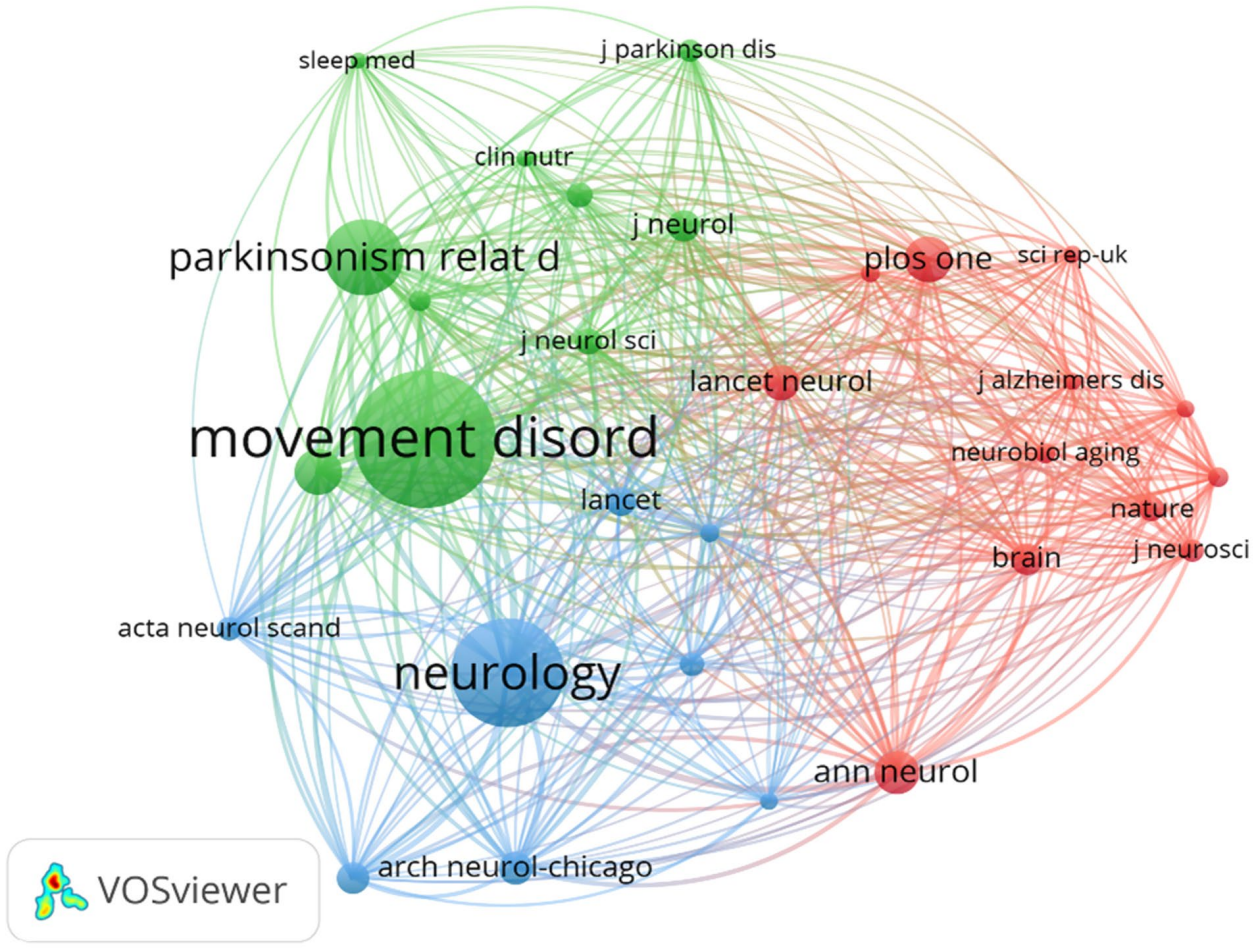
The visualization of co-cited journals for research on BMI and PD. Created with VOSviewer.

**Figure 7:**
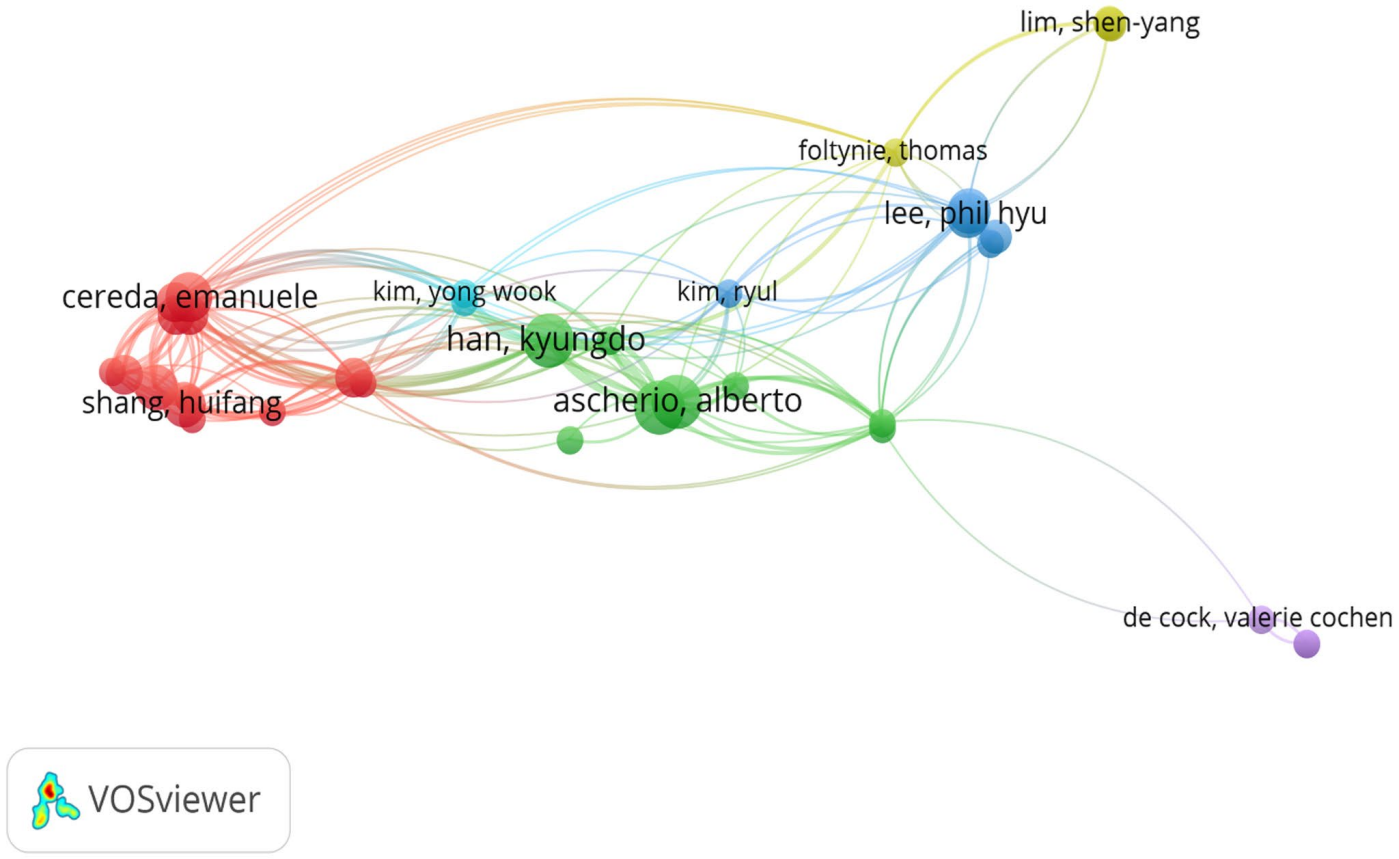
The visualization of authors for research on BMI and PD. Created with VOSviewer.

A total of 3,707 authors participated in studies on BMI and PD during the study search period. A network of co-occurring relationships was established for the study based on authors who published a minimum of five papers. Figures 6, 7 illustrates the co-occurring relationships between researchers in the literature. Each researcher is represented by a node, and the thickness of the edge between two researchers indicates the frequency with which they co-occur in the same document. The size of each node indicates the salience of the researcher, which is measured by how often they appear in the literature. Furthermore, the nodes are color-coded according to the outcomes of the cluster analysis, which categorizes them into distinct clusters that reflect thematic relationships or author groupings. The network visualization reveals each individual cluster, and Ascherio Alberto, Han Kyungdo, and Cereda Emanuele have the largest nodes because they have published the most relevant works.

**Figure 8:**
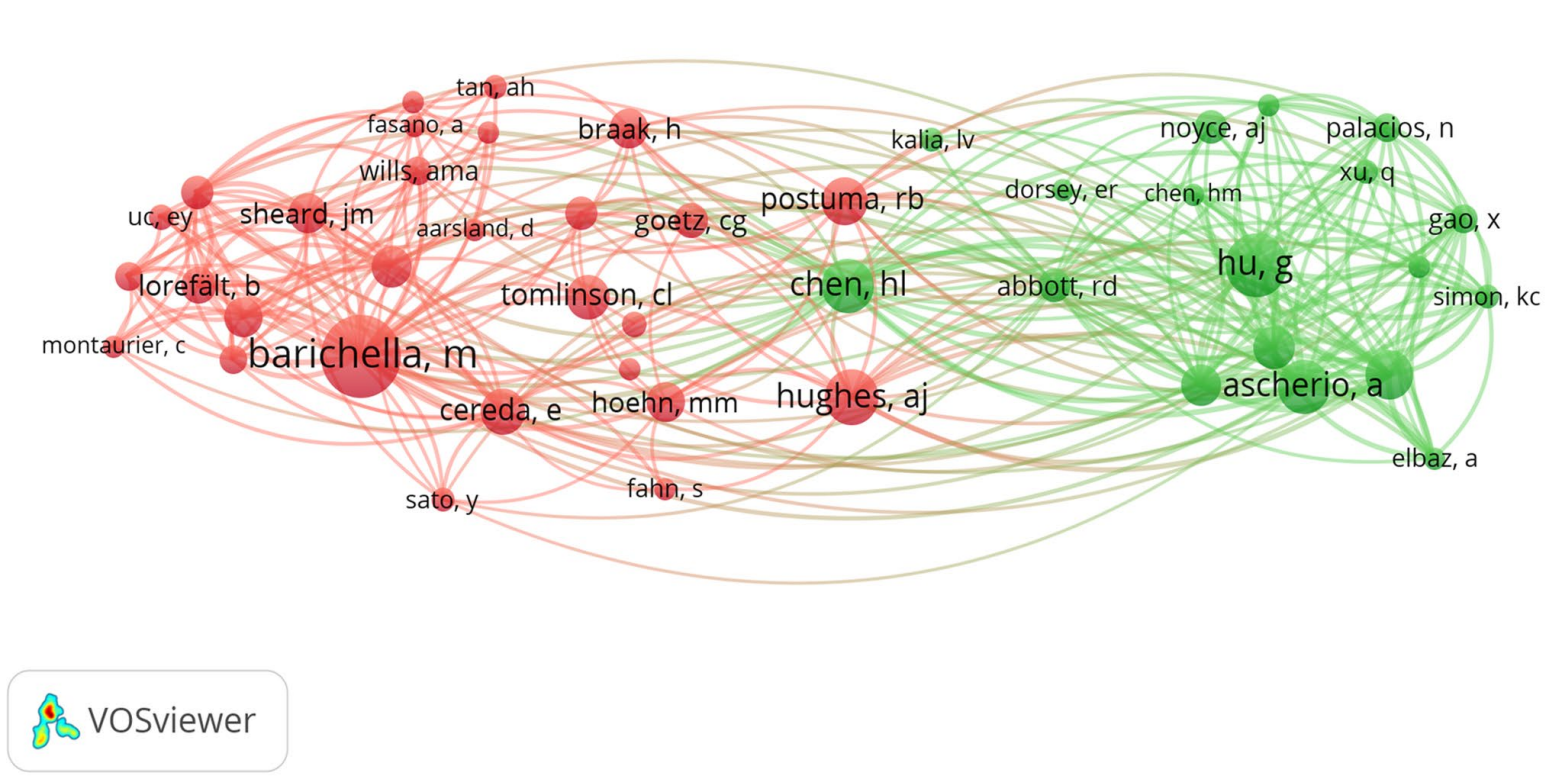
The visualization of co-cited authors for research on BMI and PD. Created with VOSviewer.

Among the top 10 authors in terms of publications, Lu Zhang published 12 papers, followed by Su Jin Chung with 11 papers (Table 4). Figure 8 illustrates the 18,168 authors who were co-cited in BMI and PD-related references, with the clustering of red nodes representing academic communities that work closely together in the field of research on BMI and its relationship with PD. Conversely, green nodes represent researchers who collaborate in other areas. Notable among the red nodes are Michela Barichella and Gang Hu. In conjunction with Table 4, Michela Barichella (*n* = 152) emerges as the most co-cited author. The scope of his research encompasses diverse domains, including PD and BMI, nutritional management, and gut flora. Noteworthy is his study in 2003, which reported that deep brain stimulation not only significantly improved dyskinesia in patients with PD, but also increased BMI (Barichella et al., 2003). In 2019, he published Gut Flora Relevant to PD, which posits that gut flora may serve as environmental regulators, contributing to inter-individual variability in clinical features. A notable finding was the lower abundance of Trichoderma spp. in patients with new-onset PD compared to healthy controls (Barichella et al., 2019). This study contributes to the existing body of research on the factors influencing BMI, including but not limited to gut flora and vitamin D, as well as nutritional management in PD patients.

**Table 4:** Top 10 authors and co-cited authors for research on BMI and PD.

Authors	Articles	Author	Citations
ZHANG L	12	barichella, m	152
CHUNG SJ	11	hu, g	109
ASCHERIO A	10	hughes, aj	94
CEREDA E	10	chen, hl	91
HAN K	9	ascherio, a	90
ZHANG Y	9	chen, h	80
BARICHELLA M	9	postuma, rb	76
CASSANI E	8	cereda, e	73
LIU Y	8	tomlinson, cl	71
PEZZOLI G	8	de lau, lml	68

### 3.6 Analysis of the co-cited references

**Figure 9:**
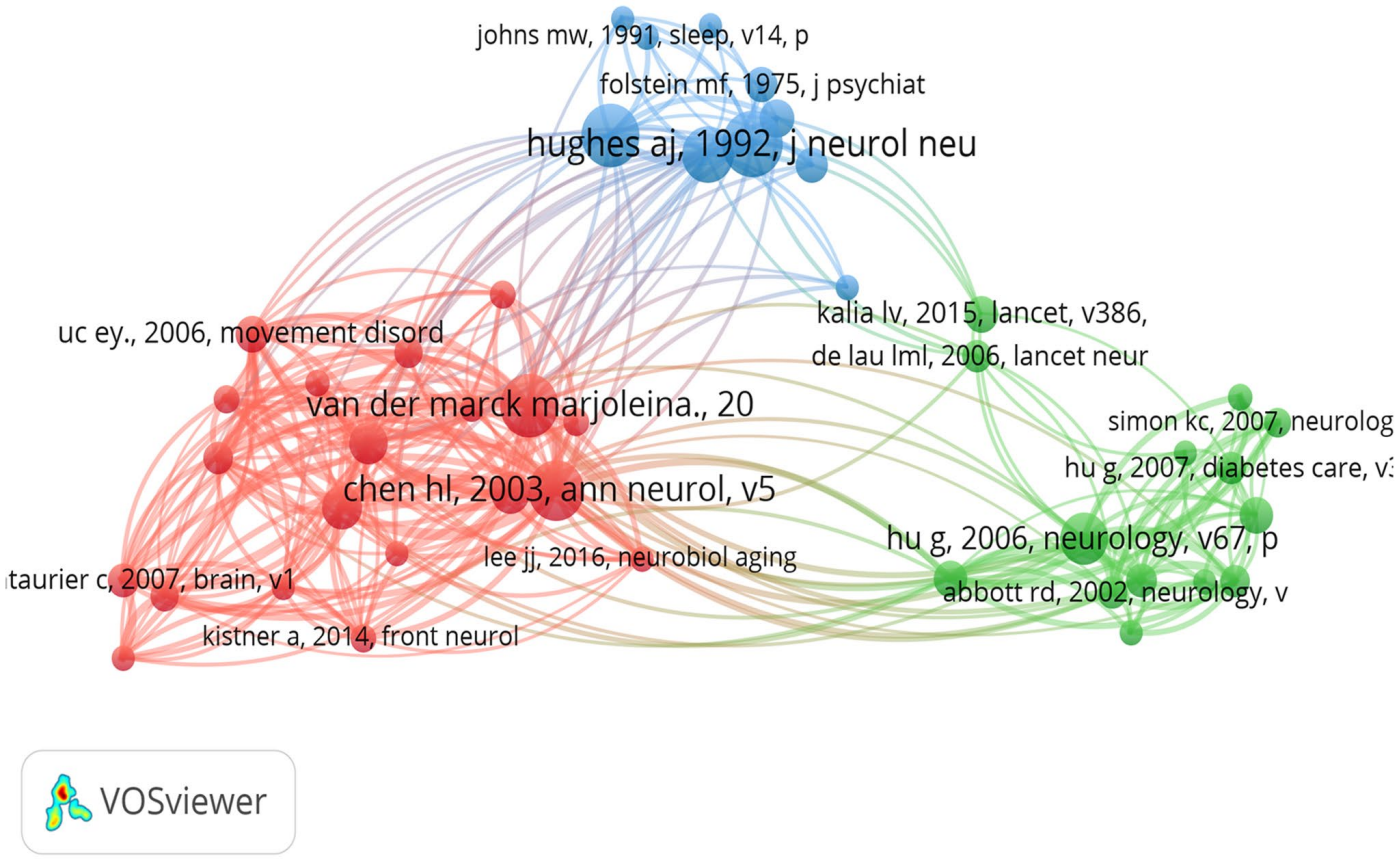
The visualization of co-cited references for research on BMI and PD. Created with VOSviewer.

A total of 23,950 citations were identified in the literature database of this paper, with 44 citations exceeding 20 co-citation frequencies, indicating a strong connection between academic works in this field. The co-citation network mapping (Figure 9) illustrates the interconnections between these influential studies. The nodes in the graph represent cited literature, and the size of the nodes is proportional to the citation frequency of the literature. The network mapping is divided into three main color clusters representing different research areas or topics, with each cluster representing a different focus of the broader research area. For instance, the red cluster, comprising the works of Hsiu-Ling Chen and Van der Marck Marjolena, focuses on weight changes in PD patients before and after diagnosis and the effect of disease progression in PD on BMI, respectively. The green cluster, which is predominantly composed of studies by Andrew J Hughes and Claire L Tomlinson, includes research by the former on the prospective diagnosis of PD from a pathological perspective (Hughes et al., 1992) and the latter on a useful tool for the dosage intensity of different anti-PD drug regimens (Jeong et al., 2020). The blue cluster primarily consists of Hoehn MM studies on the onset, progression, and mortality of PD. Hu et al. (2006) found that BMI is associated with the risk of PD. This association was found to be graded and independent of other risk factors. A significant direct association was identified between triceps skinfold thickness and PD risk, independent of BMI and other potential confounders. In addition, waist circumference and waist-to-hip ratio were found to be associated with an elevated risk of PD in never smokers. The seminal work by Bachmann et al. (2006) on potential factors for weight loss in patients with PD further underscores the intricacy and depth of research topics surrounding the correlation between BMI and PD.

### 3.7 Reference with citation bursts

**Figure 10:**
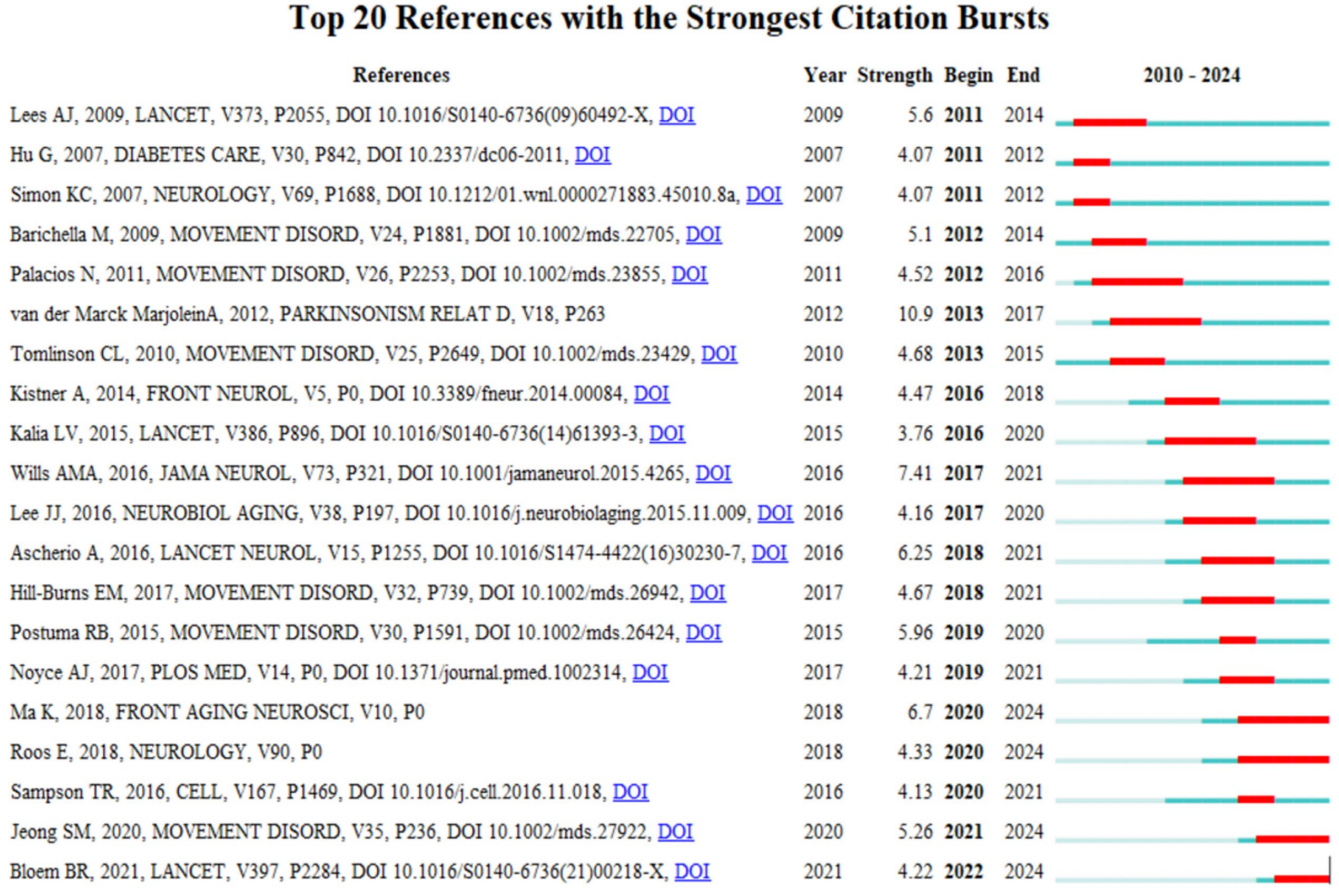
The visualization of citation bursts. Created with Bibiometrix package for R language.

In this study, a citation-surge analysis of highly cited literature in the field of PD was conducted using CiteSpace. The analysis identified 20 references with the strongest citation surges (Figure 10). These literatures garnered substantial academic attention within a designated time frame, reflecting the prevailing themes and cutting-edge trends in PD research. A notable example is van der Marck MA, in Mov Disord, “Effectiveness of multidisciplinary care for Parkinson’s disease: a randomized, controlled trial.” This randomized and controlled trial (surge intensity: 10.9, 2013–2017) is a seminal study in the field, focusing on multidisciplinary management strategies for PD and significantly advancing the integrative treatment paradigm. Secondly, the article published by Wills et al. (2016), with a surge intensity of 7.41, explored the associations between changes in BMI, Unified PD Rating Scale (UPDRS) exercise and total scores and survival of patients with PD. It examined whether there was a positive correlation between BMI at randomization and survival, revealing the association between dynamic changes in BMI and the progression of PD (Wills et al., 2016). This article is considered a milestone in the field of metabolic research. The preponderance of high surge literature was disseminated through prominent journals (e.g., LANCET, JAMA NEUROL, and CELL), and their findings have garnered extensive citations, thus becoming foundational references within the field. These literatures have not only promoted the theoretical development of PD research, but also provided important guidance for clinical practice.

### 3.8 Analysis of the keywords

**Figure 11:**
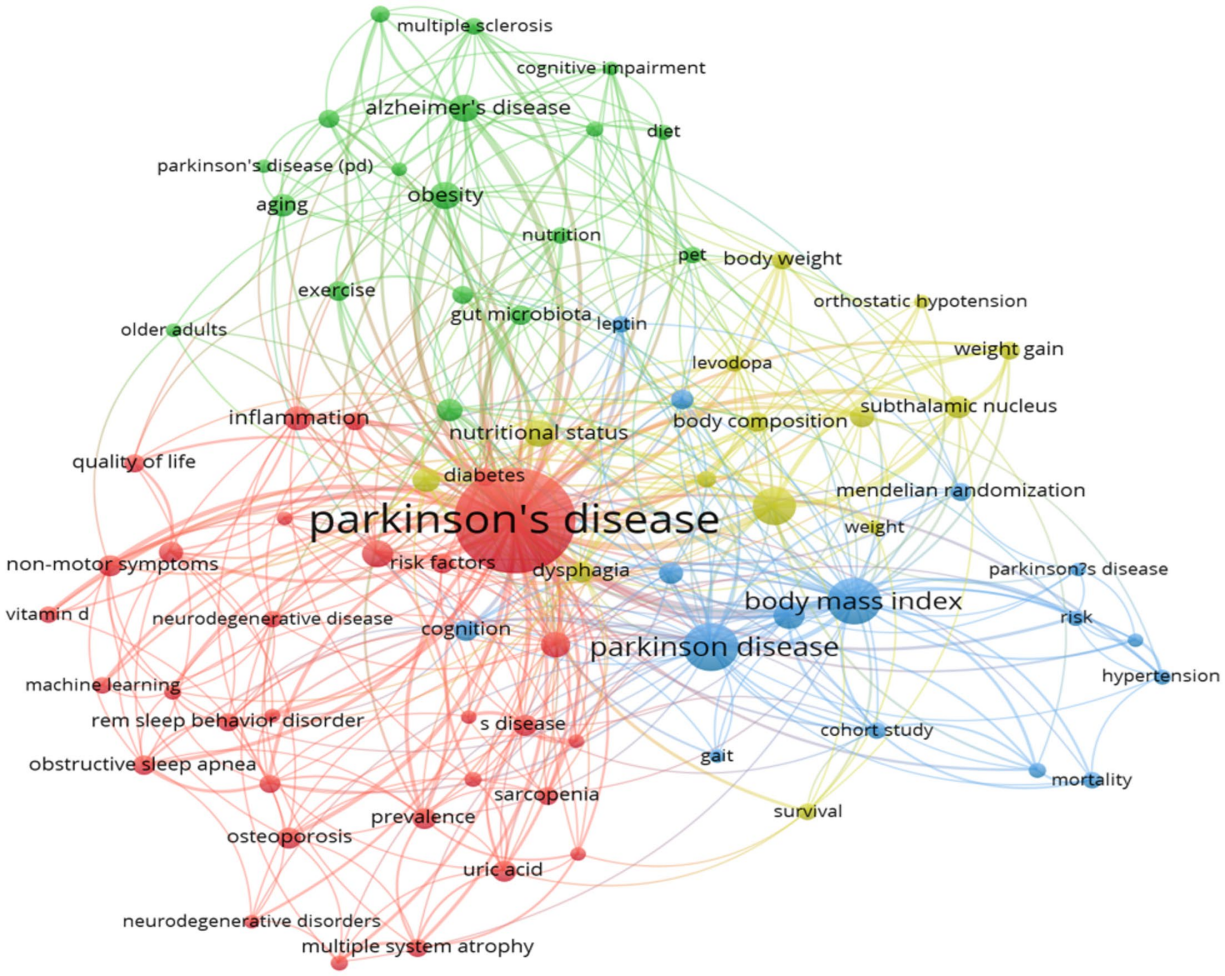
The visualization of keywords. Created with CiteSpace 6.4 R1.

In this study, the keywords of 588 documents were analyzed by co-occurrence network analysis using VOSviewer. The analysis yielded 107 keywords with a frequency of more than 10 occurrences, which were then extracted for visualization (Figure 11). Through clustering analysis, multiple clusters of research themes were identified, reflecting the core research directions and hot trends in PD and related fields. The core keywords clustered in red include “weight gain” “weight loss” “body mass index” and this cluster focuses on the metabolic changes and nutritional management of PD patients, and reveals the role of weight fluctuation (e.g., the association between weight loss and worsening of non-exercise symptoms) and BMI in disease progression. The keywords “main nutrition” and “elderly patients” indicate that nutritional intervention strategies (e.g., Mediterranean diet) for elderly patients are hot in current research. The node “energy expenditure” indicates a close relationship between BMI and weight change, suggesting that changes in metabolic rate may be a potential mechanism for weight fluctuation in PD. Constipation, a prevalent non-exercise symptom, is intimately linked to nutrient absorption and weight management. The blue clustering reflects the use of neuromodulation techniques in PD treatment, especially the deep brain stimulation, in improving motor symptoms. The term “subthalamic nucleus” signifies the thalamic nucleus as the primary target of the deep brain stimulation, while “outcomes” underscores the significance of evaluating treatment efficacy. The green clusters primarily pertain to the shared pathological mechanisms of neurodegenerative diseases, including PD and other such conditions as Alzheimer’s disease and amyotrophic lateral sclerosis. The keywords “alpha-synuclein” and “oxidative stress” suggest that protein misfolding and oxidative damage are central to the research. The yellow clusters, on the other hand, emphasize non-motor symptoms of PD, such as cognitive impairment and depression.

**Figure 12:**
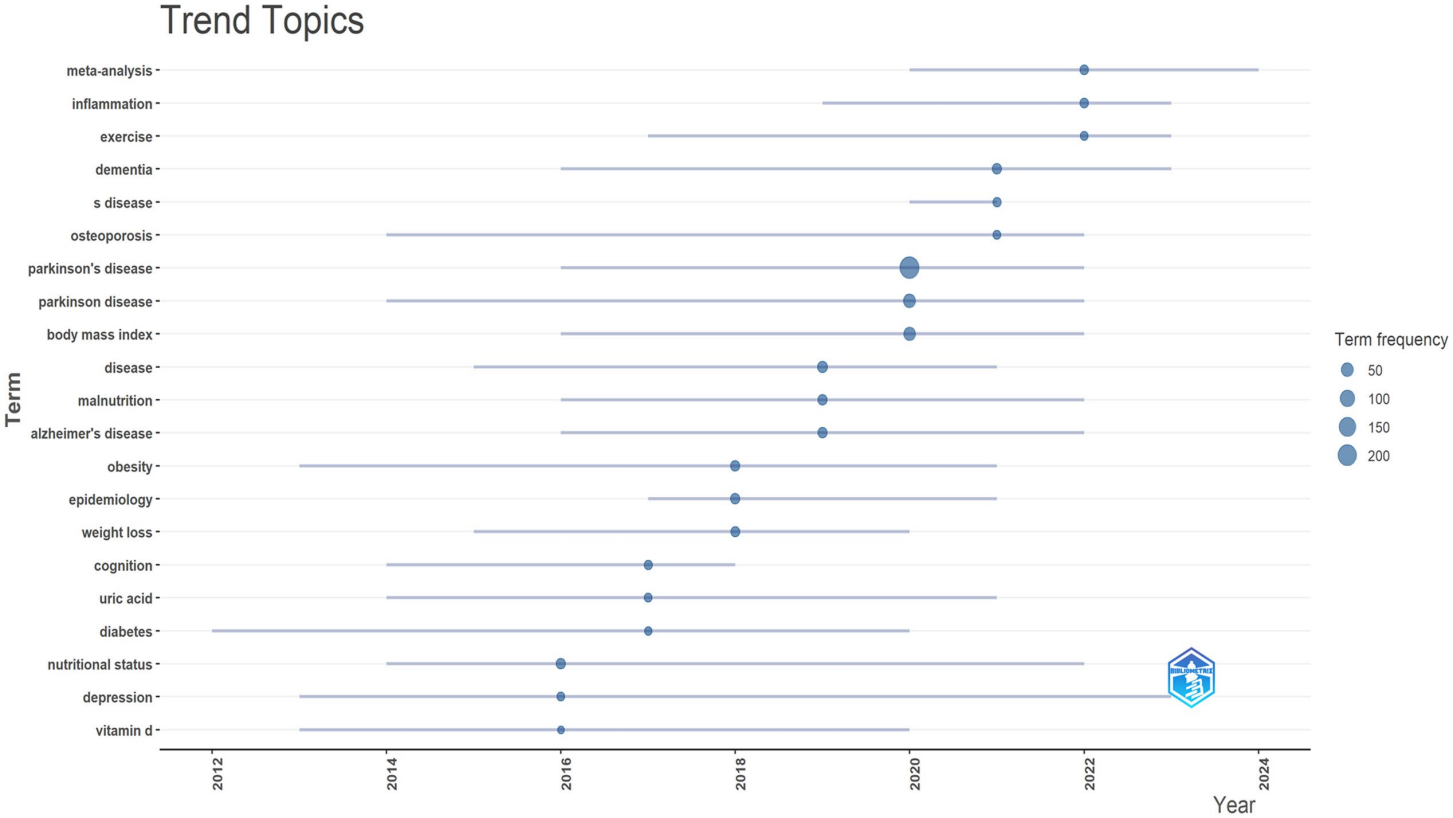
Trend topics. Created with Bibiometrix package for R language.

The trend theme analysis depicted in Figure 12 highlights the evolving research focus and emerging trends in BMI and PD studies between 2010 and 2024 (Figure 12). The early emphasis on pathological mechanisms such as “oxidative stress” and ‘dopamine’ reflects the dominance of basic research on neurodegeneration in PD. The initial emergence of the keywords “body-weight” and “physical-activity” signaled early exploration of metabolic interventions. A significant increase in the frequency of “body-mass index” and “weight-loss” in 2020 (reaching a frequency peak of 100) signaled a shift in research emphasis toward metabolic regulation and clinical relevance. Key policy drivers during this phase (such as the U. S. “Precision Medicine Initiative”) spurred the application of multi-omics technologies (e.g., gut microbiome analysis). In recent years, “quality-of-life” and “insulin-resistance” have emerged as hot topics (frequency 75–125), reflecting research expansion from symptom management toward holistic patient health and metabolic syndrome. Concurrently, the increased co-occurrence of “Parkinson-disease” and “dementia” signals the rise of cross-disease mechanism studies.

## 4 Discussion

Bibliometrics is an important tool for quantifying and analyzing the changing landscape of a particular field of study. By utilizing modern computational techniques, it generates a clear and concise knowledge map that facilitates researchers to understand the fundamentals, prevalent foci, and emerging trends in the field across time and geographic boundaries (Merigó et al., 2015). In this study, we systematically elucidated the role of BMI in the progression and treatment of Parkinson’s disease and its research trends through bibliometric analysis. Unlike traditional systematic evaluations, bibliometric analyses, by synthesizing the extant published literature, can not only reveal the historical evolution of the research field, but also predict the future direction of research, thus providing a scientific basis for clinical practice and policy formulation.

### 4.1 The mechanism by which BMI influences the progression of Parkinson’s disease

The year 2016 was a key inflection point in the field of PD and BMI research, with the number of publications exceeding 40 for the first time in that year, an increase that may be directly related to precision medicine policies and technological maturity (e.g., decreasing cost of single-cell sequencing). Single-cell sequencing allows for the discovery and characterization of cell types and their functional states, promoting research in cellular differentiation and reprogramming (Balzer et al., 2021). Consequently, the significant decrease in sequencing costs has greatly advanced PD research. This progress includes linking PD to specific genetic variants and uncovering associations of disease traits within particular cell types, which helps identify potential therapeutic targets (Smajić et al., 2022). Combining the annual publication volume, citation emergence and keyword emergence, we reveal the development trend and hotspot changes in the field of PD and BMI research. Citation bursts reflect the timing and trend of topic evolution.

Early-stage citation emergence literature focused on diagnostic criteria (e.g., Lees et al., 2009) and pharmacological treatments (e.g., Tomlinson et al., 2010) for PD, reflecting the urgent need for clinical practice guidelines at that stage. The focus of the mid-stage citation explosion gradually shifted to metabolic and microbiome studies.

Zhong and Zhang, 2025 demonstrated that early changes in BMI can predict cognitive decline and alterations in AD (Alzheimer’s disease, AD) biomarkers in PD patients. The group with BMI decline exhibited significantly reduced cerebrospinal fluid Aβ42 levels (*β* = −53.732, *p* = 0.049), suggesting BMI monitoring may serve as an early indicator of PD progression (Zhong and Zhang, 2025). A 2021 study published in Parkinsonism and Related Disorders indicated that weight loss is significantly associated with accelerated striatal dopamine degeneration (Pak et al., 2018). Additionally, reports suggest that leptin produced by adipose tissue may prevent dopaminergic neuron degeneration (Weng et al., 2007). BMI levels are closely linked to gut microbiota, with low BMI revealing profound gut microbial alterations (Rinninella et al., 2019). Small intestinal dysbiosis may explain weight loss in PD patients, where reduced triglyceride (TG) uptake is thought to result from bacterial overgrowth (Shih et al., 2024).

Although these findings provide insights into potential mechanisms linking BMI to PD progression, the precise causal pathways underlying this association require further investigation. This is especially important given the potential for reverse causality from disease-related weight loss.

### 4.2 High-potential directions in the field of precision nutrition

The surge in citations to Sampson et al. (2016) signals a shift in PD research from a single neurological mechanism to a multi-system interaction. The gut microbiota influences neurodevelopment and regulates behavior. Sampson TR’s research suggests that alterations in the human microbiome constitute a risk factor for Parkinson’s disease. Single-cell sequencing technology has made it possible to study the association of the gut microbiome with metabolic abnormalities in PD. Keywords that have emerged range from “population,” “oxidative stress” and “nutritional status.” The keywords change from “population,” “oxidative stress” and “nutritional status” to “mild cognitive impairment” and “validation.” Validation, showing to the expansion of the research focus from a focus on PD risk factors, pathomechanisms, and single motor symptoms to multidimensional symptom management. For a long time, Barichella et al. (2009) and Wills et al. (2016) have committed themselves to and focused their work on Parkinson’s disease research. The citation surge of Barichella et al. (2009) and Wills et al. (2016) revealed the association between changes in BMI dynamics and disease progression, driving the development of metabolic intervention strategies. Analyzed in conjunction with historical events, the surge in post-2016 publications can be attributed to breakthroughs in multi-omics technology and deepening interdisciplinary collaborations.

Jeong et al. (2020) found an increased risk of PD onset in the underweight group compared to the normal weight group, while a decreased risk of PD onset was observed in the obese and severely obese groups. For patients with severe diabetes, the steepest slope of decrease in PD risk was observed with increasing body mass index (Jeong et al., 2020). A Korean study analyzing weight changes and survival outcomes in PD patients found that, compared to sustained normal BMI or overweight status, a persistent underweight status, a decline in BMI to underweight, or weight gain from underweight to normal/overweight were all associated with an increased mortality risk (Yoon et al., 2024). Therefore, individualized weight management plans should be developed based on each patient’s baseline BMI. It is recommended that patients maintain a weight ranging from normal to obese, or achieve a slight weight gain—specifically, a target increase in BMI of 2 kg/m^2^ (Yoon et al., 2024). Additionally, a dynamic monitoring strategy is advised, involving regular combined assessments of BMI and waist circumference alongside inflammatory markers (e.g., IL-6, TNF-*α*) to improve predictive accuracy (Zhong and Zhang, 2025).

The relationship between dietary interventions, exercise therapy, and BMI is a central topic in obesity management and metabolic health research. These three interact with each other and work together in energy balance, which in turn regulates body weight and body fat distribution. Studying the relationship is important for developing scientific and effective health intervention strategies.

The non-motor symptoms of patients with PD play an important role in their nutrient metabolism, and dysphagia can lead to impaired nutritional status and fluid balance. Gastrointestinal issues (such as diminished sense of smell, taste disorders, dry mouth, orofacial tremors, difficulty chewing, dysphagia, gastroparesis, and small bowel motility disorders) along with reduced physical flexibility may all interfere with normal eating or food digestion. Premature satiety often leads to insufficient energy intake. Metabolic changes resulting from neuroendocrine dysfunction in peripheral and/or hypothalamic appetite signaling pathways may represent the underlying mechanism of PD. Furthermore, depression, cognitive impairment, and apathy may diminish the desire to eat, while increased energy demands due to movement disorders further elevate overall energy expenditure. Additionally, as gastrointestinal side effects from disease-progression therapy gradually worsen, they may make obtaining or consuming food more difficult (Henderson et al., 2025).

In the management of BMI in patients with PD, optimizing pharmacological treatment to improve motor and non-motor symptoms is crucial, but it needs to be combined with scientific nutritional intervention strategies to ensure balanced nutrition and prevent abnormal weight fluctuations. Levodopa, as one of the primary long-term treatments for Parkinson’s disease, may exacerbate existing gastrointestinal symptoms in patients with single or repeated administration. Concurrently, delayed gastric emptying in Parkinson’s patients inhibits the normal absorption of levodopa or dopamine agonists, creating a vicious cycle (Perez-Pardo et al., 2017). This hormonal disturbance may trigger a type of lipolytic cycle due to increased basal metabolic rate and weight loss (Sirtori et al., 1972). Despite the absence of a comprehensive understanding of the mechanisms underlying the association between obesity and PD risk, it has been demonstrated that dopamine plays a significant role in regulating food intake. The lower availability of dopamine D receptors in the striatum of obese individuals may contribute to the development of eating disorders and obesity. Furthermore, obesity has been demonstrated to augment the risk of PD by means of increasing oxidative stress and dopaminergic neuronal death (Hu et al., 2006). It is noteworthy that obesity and gut microbiota dysbiosis share a close bidirectional association. Obesity is often accompanied by alterations in gut microbial composition, including reduced diversity and increased Firmicutes/Bacteroidetes ratio, which may further impact energy metabolism (Ley et al., 2006). Simultaneously, gut microbiota participate in regulating brain-gut axis function, influencing the synthesis and signaling of neurotransmitters such as dopamine, thereby indirectly modulating feeding behavior and weight regulation (Perez-Pardo et al., 2017). Therefore, in Parkinson’s disease weight management, adopting precision nutrition interventions targeting the gut microbiota—such as appropriately increasing dietary fiber, prebiotics, and specific probiotics to optimize microbial composition, reduce systemic inflammation, and improve metabolic status—not only aids in maintaining healthy weight but may also exert positive regulatory effects on dopaminergic neural function.

Other strategies involve optimizing the pharmacokinetics of levodopa to minimize interference from dietary proteins, enhancing gastrointestinal function—including the management of dysphagia and constipation—and preventing or correcting deficiencies in micronutrients and vitamins. For instance, it is recommended to ensure a daily protein intake of at least 1.2 g/kg, prioritizing high-quality sources rich in vitamin B12 and dietary fiber, such as fish, eggs, legumes, and lean meats. Caloric intake should be adjusted according to body weight status to achieve or maintain a normal weight in underweight or obese patients (Xie et al., 2025). In early disease stages, a balanced Mediterranean-style diet is advised, while patients in advanced stages may benefit from protein-adjusted diets with moderate protein restriction (Barichella et al., 2009). These nutritional interventions show considerable promise for clinical application.

### 4.3 A research gap assessment of digital health tools is hereby presented

The 2021–2024 citation burst focuses on precision medicine (e.g., Bloem et al., 2021) and digital health technology (e.g., Jeong et al., 2020), reflecting the trend of PD research toward individualization and intelligence. Leveraging AI to analyze vast amounts of biomedical data can significantly shorten the drug development cycle and reduce costs. The rapid advancements in artificial intelligence (e.g., ChatGPT, DeepSeek) are poised to provide novel tools for predictive modeling. The rapid advancement of artificial intelligence (such as ChatGPT and DeepSeek) has provided entirely new tools for predictive modeling. In precision medicine, AI can integrate an individual’s genomic data, lifestyle information, and medical history to tailor optimal treatment and prevention plans for patients. Consequently, future research may focus on digital therapeutics (such as FDA-approved AI prescription systems), enabling a dual growth trajectory of “technology-clinical” convergence.

Bloem et al. (2021) screened 100 publications from 2017 to 2020 that were related to clinical diagnostic treatments for patients with PD and other related literature were comprehensively analyzed to summarize prognostic, multidisciplinary management protocols for different PD patients. They mentioned remote medical monitoring through methods such as body-worn or home-based sensors, smartphone applications, or keystroke dynamics analysis. This approach allows for the objective and continuous assessment of a patient’s condition at home, thereby facilitating timely adjustments to treatment and rehabilitation plans (Bloem et al., 2021). A comprehensive analysis of 6,800,601 PD-naïve individuals (age ≥40 years) was conducted using the National Health Insurance Service database in South Korea. The risk ratios of PD were assessed using Cox proportional risk regression, with stratification by diabetes status. The findings indicated that underweight and diabetes mellitus were associated with an elevated risk of developing PD, with the effect of underweight being more pronounced in diabetic patients (Jeong et al., 2020).

The association between low protein intake and an increased risk of sarcopenia and reduced muscle strength in patients with PD highlights the importance of regular nutritional monitoring as part of comprehensive PD management and sarcopenia prevention. It is essential to monitor dietary intake, with particular emphasis on protein consumption, while also accounting for other nutritional factors such as daily caloric intake, overall diet quality, and relevant clinical variables. An AI-powered comprehensive monitoring system can efficiently process and synthesize large-scale monitoring data, enabling timely dietary reminders and personalized training recommendations to help preserve muscle strength and enhance physical function. Such integrated approaches hold promise for improving the quality of life in patients with Parkinson’s disease (Lima et al., 2025).

The BMI has been demonstrated to be closely related to the gut microbiota, and low BMI levels have been shown to result in significant alterations to the gut microbiota (Rinninella et al., 2019).

There is evidence that patients with PD generally exhibit a lower BMI compared to healthy individuals of the same age, and that low BMI is associated with low bone mineral density, both of which are major risk factors for hip fracture (Sato et al., 2018). Potential factors contributing to weight loss in patients with PD include decreased sense of smell, hand-mouth coordination deficits, dysphagia, intestinal hypokinesia, depression, and decreased reward processing in the limbic region of the dopaminergic midbrain. Consequently, Bachmann and Trenkwalder (2006) have proposed the implementation of monthly weight monitoring of PD patients during disease progression and nutritional supplementation, such as adequate vitamin D and calcium, to mitigate the risk of hip fracture and to enhance bone mineral density post-study. Additionally, flexible dietary schedules and meal-assisted strategies are of particular importance for PD patients in nursing facilities, as the timing of their diets may be affected by motor impairments and “off periods” (Bachmann and Trenkwalder, 2006). The high frequency of the keywords “quality of life” and “epidemiology” suggests a shift in focus from the disease itself to the overall health and quality of life of patients. Healthy dietary patterns, such as the antioxidant-rich Mediterranean diet, may reduce Parkinson’s disease risk by alleviating oxidative stress, lowering inflammation, and improving mitochondrial function (Feng et al., 2020).

Marco Sicadengren have systematically collected relevant literature and proposed that wearable devices (e.g., inertial sensors) have the capacity to collect data over extended periods of time and have the potential to facilitate the monitoring of patients with PD, providing relevant information about their motor status, improving patient-physician interactions, influencing treatment decisions, and ultimately improving the overall health of patients (Sica et al., 2021; Maetzler et al., 2013). The framework of “precision medicine” proposed by Bloem et al. (2021) in LANCET emphasizes the importance of individualized treatment, but its application to digital health tools needs to be further explored.

A substantial corpus of highly cited literature in the domain of metabolic and nutritional research has emerged, illuminating the association between dynamic fluctuations in BMI and the progression of PD. This has consequently given rise to the formulation of metabolic intervention strategies (Sheard et al., 2014). The majority of current studies have focused on traditional interventions. The application of digital health tools (e.g., wearables to monitor energy metabolism) in PD management shows great promise, but there are significant research gaps. For instance, the study by Sampson et al. (2016) published in CELL revealed for the first time the mechanism by which the gut microbiome affects motor symptoms in PD through neuroinflammation, opening up a new direction in the study of the microbe-gut-brain axis (Kacprzyk et al., 2022). However, translating this mechanism into a clinically usable digital tool remains a challenge. To address this knowledge gap, future research should prioritize the development of digital health tools capable of real-time energy metabolism monitoring and dynamic adjustment of nutritional programs.

In the direction of policy synergy, future research can learn from the U. S. “All of Us” program to develop an open-source data ecosystem, establish a medical multimodal database (e.g., patient lifecycle health data), and then integrate multi-omics data (genome, metabolome, and microbiome) to build personalized nutrition models.

### 4.4 Limitations in BMI management for PD patients

Although analysis of previous studies suggests BMI monitoring may serve as an early indicator of PD progression, it has limitations. Particularly in elderly PD patients, it may mask important changes in body composition, such as sarcopenic obesity. The BMI formula is calculated by dividing weight by the square of height (kg/m^2^). It cannot distinguish between: Fat Mass and Fat-Free Mass (especially muscle mass), nor the distribution of fat (subcutaneous fat vs. visceral fat, the latter posing greater health risks). Some patients may exhibit significant muscle loss, known as sarcopenia, despite having normal or elevated BMI. This phenomenon is particularly common in PD patients and is associated with motor dysfunction, inadequate protein intake, and chronic inflammation. Therefore, clinical practice still requires the integration of other body composition measurement indicators (such as grip strength, gait speed, bioelectrical impedance analysis, etc.) to comprehensively assess nutritional and muscular status (Barichella et al., 2019).

## 5 Conclusion

As the first bibliometric analysis, a detailed overview of research trends on the correlation between BMI and PD from 2010 to 2024 is provided. The steady increase in the number of publications suggests an increasing emphasis on research in this area by scholars worldwide, with China and the United States leading the field. Early research has focused on the diagnosis, pathology, and epidemiology of PD, and metabolic intervention strategies. Emerging frontiers include the gut-brain axis, and smart AI monitoring, demonstrating new therapeutic potential. This study emphasizes that future directions should focus on the use of multidisciplinary integrated therapies in research and treatment development, advancing national understanding of the relationship between BMI and PD patients, and creating therapies that effectively slow disease progression and reduce mortality by adjusting BMI in PD patients.

## 6 Strength and limitations

The strength of this bibliometric analysis lies in its systematic collection of literature on the correlation between brain-machine interfaces BMI and PD over the 15-year period from 2010 to 2024, coupled with the effective visualization and analysis of large-scale datasets using tools such as VOSviewer and CiteSpace. These approaches provide valuable insights into research trends, hotspots, and influential works in the field. However, the study’s reliance solely on the Web of Science Core Collection may limit the comprehensiveness of the analysis. Additionally, bibliometric methods inherently favor more recent and highly cited publications, potentially overlooking some seminal early-stage studies. Future research should consider integrating multiple databases (e.g., Scopus, PubMed) to enhance data inclusivity and accuracy. Expanding the scope to incorporate clinical studies would also offer a more holistic perspective on the field and facilitate the translation of basic research findings into clinical applications.

## Data Availability

The original contributions presented in the study are included in the article/supplementary material, further inquiries can be directed to the corresponding authors.
